# A Curious Cyst Beyond the Thorax: A Rare Case of Extrathoracic Bronchogenic Cyst Over the Shoulder in a Child

**DOI:** 10.7759/cureus.99442

**Published:** 2025-12-17

**Authors:** Khushboo Jha, Shishir Kumar, Radhika Narayan, Minakshi Mishra

**Affiliations:** 1 Department of Pathology, Tata Main Hospital, Jamshedpur, IND; 2 Department of General Surgery, Tata Main Hospital, Jamshedpur, IND

**Keywords:** bronchogenic cyst, congenital malformation, extrathoracic cyst, pediatric cystic lesion, supraclavicular swelling

## Abstract

Bronchogenic cysts are rare congenital malformations originating from abnormal budding of the tracheobronchial tree during embryogenesis. While typically found in the mediastinum or lungs, extrathoracic presentations, particularly in the scapular or shoulder region, are exceedingly rare. We report the case of a three-year-old female with a cystic lesion over the left supraclavicular area that was histopathologically diagnosed as a bronchogenic cyst. This case underscores the importance of including bronchogenic cysts in the differential diagnosis of pediatric cystic masses outside the thorax. Surgical excision remains both diagnostic and curative.

## Introduction

Bronchogenic cysts (BCs) are congenital anomalies that arise from abnormal budding of the ventral foregut and tracheobronchial tree between the third and sixth week of embryonic life [1]. These cysts are typically lined by ciliated columnar epithelium, often accompanied by cartilage, smooth muscle, and mucous glands like bronchial wall tissue [2].

BCs are usually located in the mediastinum, especially in the paratracheal or carinal regions, and account for 10% to 15% of all mediastinal tumors and over half of all mediastinal cysts [3]. Rarely, they may occur in ectopic extrathoracic sites, including cervical, periscapular, subcutaneous, or even intra-abdominal locations [4]. These atypical locations are thought to result from the migration of embryonic remnants during development.

Scapular or shoulder-region BCs are extremely rare, with only a limited number of cases documented in the literature. They are often misdiagnosed preoperatively as lipomas, lymphangiomas, dermoid cysts, or other soft tissue masses due to overlapping clinical and radiological features [5,6].

Here, we present a rare case of a pediatric patient with a BC located in the left suprascapular shoulder region. We emphasize the importance of considering BCs in differential diagnoses for cystic lesions in unusual locations and highlight the role of surgical excision and histopathological evaluation in confirming the diagnosis.

## Case presentation

A three-year-old female child presented to the Paediatric Surgery Outpatient Department with a history of a progressively enlarging swelling over the left shoulder region. The swelling had been first noticed by the parents at around one year of age and was described as soft and painless. Over the past several months, the child had experienced multiple self-limiting episodes of upper respiratory tract infections, but there was no associated fever, local redness, discharge, or history of trauma to the site.

On clinical examination, the swelling was noted over the left supraclavicular region, just lateral to the base of the neck. It was approximately 2 × 1.5 cm in size, soft in consistency, mobile, non-tender, and cystic to palpation, with no signs of local inflammation or regional lymphadenopathy. The child was otherwise healthy and developmentally appropriate for her age. Systemic examination was unremarkable.

An initial high-resolution ultrasonography (HRUSG) of the left shoulder and scapular region revealed a well-defined, oval, hypoechoic, non-vascular lesion measuring approximately 22 × 7 mm in the superficial soft tissue plane. The radiologist provided a provisional diagnosis of a lipoma or epidermoid cyst based on its morphology and echogenicity.

To further characterize the lesion, fine-needle aspiration cytology (FNAC) was performed. The aspirate yielded yellowish fluid, and the smear showed the presence of numerous cystic macrophages, anucleate squamous cells, and a few benign squamous epithelial cells in a predominantly acute inflammatory background. No granulomas or malignant cells were noted. These findings were suggestive of a benign inflamed cyst, but no definitive diagnosis could be established at this point.

**Figure 1:**
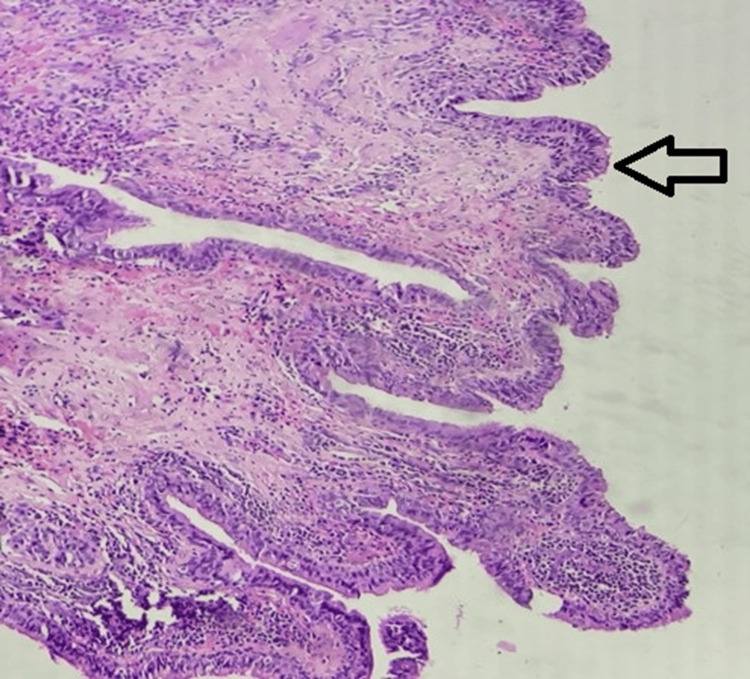
Hematoxylin and eosin, 40× view, the arrow shows the cyst cavity lined by pseudostratified ciliated columnar lining epithelium.

Given the persistence of the lesion, parental concern, and inconclusive imaging/cytology findings, the decision was made to proceed with surgical excision for both diagnostic clarification and definitive treatment. Intraoperatively, a well-encapsulated, unilocular cystic lesion was identified within the subcutaneous tissue plane. The cyst was not adherent to surrounding structures, and there were no signs of intrathoracic extension. The lesion was meticulously dissected and excised in toto. Microscopic evaluation of the cyst wall showed it to be lined by pseudostratified columnar epithelium with focal ciliation (Figure 1). The wall lacked any dermal appendages and contained areas of subepithelial inflammation. These findings were diagnostic of a BC.

Given the unusual extrathoracic location, a contrast-enhanced CT (CECT) of the thorax was performed postoperatively to evaluate for any associated mediastinal or intrathoracic bronchopulmonary foregut malformations. The CT scan was unremarkable, with no residual or concurrent cystic lesions noted in the mediastinum or lungs.

The postoperative course was uneventful. The child was discharged on postoperative day two with advice for regular follow-up. At the three- and six-month follow-up, there was no evidence of recurrence, and the surgical site had healed well.

## Discussion

BCs are rare congenital anomalies that arise from aberrant budding of the tracheobronchial tree from the primitive foregut during the third to sixth week of gestation. These cysts are typically located in the mediastinum and lung parenchyma due to their embryological origin, with over 85% occurring within the thoracic cavity. However, ectopic extrathoracic locations, including the cervical region, scapular area, subcutaneous tissue, and even intra-abdominal sites, have been infrequently reported and remain clinical curiosities.

The embryologic basis of extrathoracic BCs lies in the abnormal migration and sequestration of embryonic tracheobronchial tissue along non-typical developmental lines [6]. During early embryogenesis, the ventral portion of the foregut gives rise to the laryngotracheal diverticulum, which further differentiates into the trachea, bronchi, and lungs. If a portion of this developing respiratory epithelium gets pinched off and displaced due to abnormal budding or fusion defects, it can result in ectopic BC formation in distant sites, such as the shoulder or scapular region.

Histologically, BCs are characterized by a cyst wall lined with ciliated pseudostratified columnar epithelium, sometimes accompanied by smooth muscle, cartilage, and mucous glands, mirroring normal bronchial wall structure [7]. This histological composition is a key diagnostic criterion that differentiates BCs from other congenital cystic lesions such as dermoid, epidermoid, or branchial cleft cysts, which have distinct epithelial linings and contents.

In the present case, the cyst’s unusual supraclavicular location initially led to the diagnosis of a benign lipoma based on HRUSG. FNAC failed to provide a definitive diagnosis, as it revealed inflammatory and squamous elements suggestive of a non-specific infected cyst. This aligns with findings in previous literature, where preoperative diagnosis of extrathoracic BCs remains challenging due to their rarity and non-specific imaging features (Table 1) [8].

**Table 1:** Differential diagnosis of cystic lesions in the shoulder/supraclavicular region.

Differential diagnosis	Typical location	Clinical features	Key distinguishing points
Branchial cleft cyst	Along the anterior border of the sternocleidomastoid muscle	Soft, fluctuant, non-tender mass	Lateral neck; may become infected
Lymphangioma (cystic hygroma)	Posterior triangle/supraclavicular	Soft, compressible, transilluminant	Multiloculated; present early in life
Epidermoid/Dermoid cyst	Skin/Subcutaneous tissue	Firm, slow-growing	Keratin (epidermoid) or adnexal structures (dermoid)
Thyroglossal duct cyst	Midline neck	Moves with swallowing/tongue protrusion	Midline; from thyroglossal tract
Lipoma	Subcutaneous tissue	Soft, mobile, non-tender	Solid fatty lesion; not cystic

None of these differentials typically displays the respiratory epithelial lining, which remains the hallmark of BCs. Thus, histopathology is essential for diagnosis, especially in atypical locations where imaging and cytology are inconclusive.

Although most BCs are benign and asymptomatic, complications can arise, including secondary infection, compression of nearby structures, fistula formation, and, in rare cases, malignant transformation (e.g., adenocarcinoma, squamous cell carcinoma) [9,10]. Therefore, complete surgical excision is strongly recommended even for asymptomatic lesions, both to establish a diagnosis and prevent future morbidity.

Postoperative imaging, such as CECT thorax, as done in this case, is prudent to rule out associated mediastinal remnants or coexisting bronchopulmonary malformations, especially given the shared embryological origin. Literature suggests that recurrence following complete excision is rare, and long-term outcomes are excellent [11].

Thus, the current case adds to the growing, albeit limited, body of literature on extrathoracic BCs and emphasizes the need for a high index of suspicion when evaluating cystic masses in unusual anatomical sites in pediatric patients.

## Conclusions

BCs, though typically intrathoracic, may rarely present in extrathoracic locations such as the shoulder. Such cases pose diagnostic challenges and require high suspicion, especially in pediatric patients with cystic lesions of unknown origin. Imaging and cytology may provide initial clues, but definitive diagnosis rests on histopathology. Surgical excision remains the cornerstone of management. Clinicians should consider BCs in the differential diagnosis of pediatric soft tissue swellings in unusual locations.
